# Impact of Marker Ascertainment Bias on Genomic Selection Accuracy and Estimates of Genetic Diversity

**DOI:** 10.1371/journal.pone.0074612

**Published:** 2013-09-05

**Authors:** Nicolas Heslot, Jessica Rutkoski, Jesse Poland, Jean-Luc Jannink, Mark E. Sorrells

**Affiliations:** 1 Department of Plant Breeding and Genetics, Cornell University, Ithaca, New York, United States of America; 2 USDA-ARS, R.W. Holley Center for Agriculture and Health, Cornell University, Ithaca, New York, United States of America; 3 USDA-ARS, Northern Plains Area, Center for Grain and Animal Health Research, Hard Winter Wheat Genetics Research Unit, Manhattan, Kansas, United States of America; 4 Limagrain Europe, Chappes, France; Harbin Institute of Technology, China

## Abstract

Genome-wide molecular markers are often being used to evaluate genetic diversity in germplasm collections and for making genomic selections in breeding programs. To accurately predict phenotypes and assay genetic diversity, molecular markers should assay a representative sample of the polymorphisms in the population under study. Ascertainment bias arises when marker data is not obtained from a random sample of the polymorphisms in the population of interest. Genotyping-by-sequencing (GBS) is rapidly emerging as a low-cost genotyping platform, even for the large, complex, and polyploid wheat (*Triticum aestivum* L.) genome. With GBS, marker discovery and genotyping occur simultaneously, resulting in minimal ascertainment bias. The previous platform of choice for whole-genome genotyping in many species such as wheat was DArT (Diversity Array Technology) and has formed the basis of most of our knowledge about cereals genetic diversity. This study compared GBS and DArT marker platforms for measuring genetic diversity and genomic selection (GS) accuracy in elite U.S. soft winter wheat. From a set of 365 breeding lines, 38,412 single nucleotide polymorphism GBS markers were discovered and genotyped. The GBS SNPs gave a higher GS accuracy than 1,544 DArT markers on the same lines, despite 43.9% missing data. Using a bootstrap approach, we observed significantly more clustering of markers and ascertainment bias with DArT relative to GBS. The minor allele frequency distribution of GBS markers had a deficit of rare variants compared to DArT markers. Despite the ascertainment bias of the DArT markers, GS accuracy for three traits out of four was not significantly different when an equal number of markers were used for each platform. This suggests that the gain in accuracy observed using GBS compared to DArT markers was mainly due to a large increase in the number of markers available for the analysis.

## Introduction

Genomic selection (GS) is a new marker assisted selection method based on the simultaneous use of whole-genome molecular markers to estimate breeding values for quantitative traits [1]. GS can accelerate the breeding cycle and increase genetic gain per unit time beyond what is possible with phenotypic selection [2]. Reviews are available on the application of GS to plant breeding [3].

Key to implementing GS is the availability of inexpensive whole-genome genotyping. One such recently developed platform is Genotyping-by-Sequencing (GBS) [4]. Using advances in next generation sequencing technologies, this approach uses sequencing of multiplexed, reduced-representation libraries constructed using restriction enzymes to obtain single nucleotide polymorphism (SNP) data. The multiplexed libraries are sequenced on a single run of a massively parallel sequencing platform. GBS has very low per sample costs; an ideal situation for GS in applied programs. GBS has been used with good results for GS in wheat [5] and cassava [6]. GBS has the advantage that markers are discovered using the population to be genotyped, thus minimizing ascertainment bias. GBS typically generates a very large numbers of markers but with a high rate of missing data because genomic fragments in the library are sequenced at low depth leading to some fragments having zero coverage in some individuals.

Ascertainment bias is introduced whenever marker data is not obtained from a random sample of the polymorphisms in the population of interest. It is a sampling bias. For example, the preferential sampling of SNPs at intermediate frequencies will result in a distribution of allelic frequencies that is different compared to the expectation for a random sample. This type of biased sampling can also result from the use of a small number of lines in the SNP discovery process. This increases the frequency of the most commonly polymorphic loci and eliminates markers for loci that are less polymorphic in the screening panel. Consequently, estimates of population genetic parameters, allele frequency distribution and linkage disequilibrium can be biased [7], [8]. The effects of ascertainment bias and marker platform on genetic relationships have been studied in plants and found to have complex effects on measures of diversity and relationships between lines [9]–[11] that are not easily corrected.

A number of cereals are characterized by complex and large genome sizes (e.g. 16 Gb for wheat *Trititicum aestivum L.*). The predominant marker platform for whole-genome genotyping in wheat has been diversity array technology (DArT) [12]–[14]. DArT was developed as a hybridization-based solution, which uses a microarray platform to detect restriction site polymorphism using methylation sensitive restriction enzymes [12]. DArT generates whole-genome genotypes by scoring the presence versus absence of DNA fragments hybridized to a microarray in a reduced representation library generated from samples of genomic DNA.

DArT markers were used for most of the recent investigations concerning cereals genetic diversity and for initial studies on GS [15]–[17]. However, it is not known if diversity should be re-assessed using marker platforms subject to less ascertainment bias. In addition if reports suggest that GBS gives good results for GS in wheat [5], it is not known whether that difference is due to the large increase in the numbers of markers available or to differences between the platforms. Our objectives were to quantify the differences between the DArT and GBS marker platforms for population genetics metrics and GS accuracy in winter wheat, to determine if the same number of GBS markers can deliver prediction accuracies significantly different than DArT, and to determine whether any accuracy difference can be explained by either ascertainment bias, or non-random marker distribution across the genome.

## Results

### Diversity Analysis

**Figure 1:**
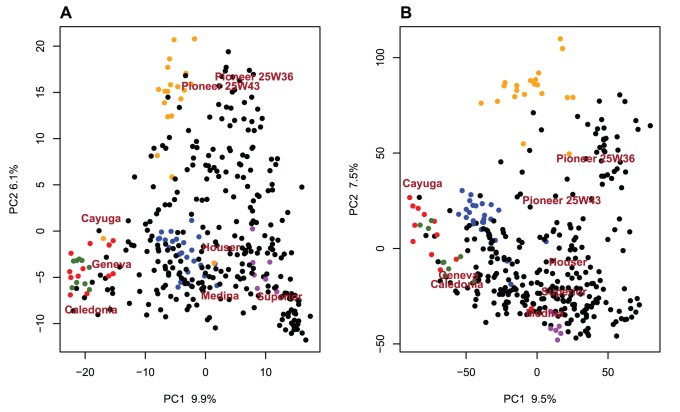
PCA plots for respectively all DArT markers (A) and all GBS markers available (B). A few large full-sibs families are color coded. (blue: Pioneer 2737W/Geneva, orange : Pioneer 2737W/Cayuga, green: Coker 8427 /AC Ron, purple: Diana/NY80095-6, red: Cayuga/Caledonia). Full-sibs are lines with the same both parents. Some of the important lines in the breeding program are indicated by their name on the plot to allow a comparison of the two PCA plots.

**Figure 2:**
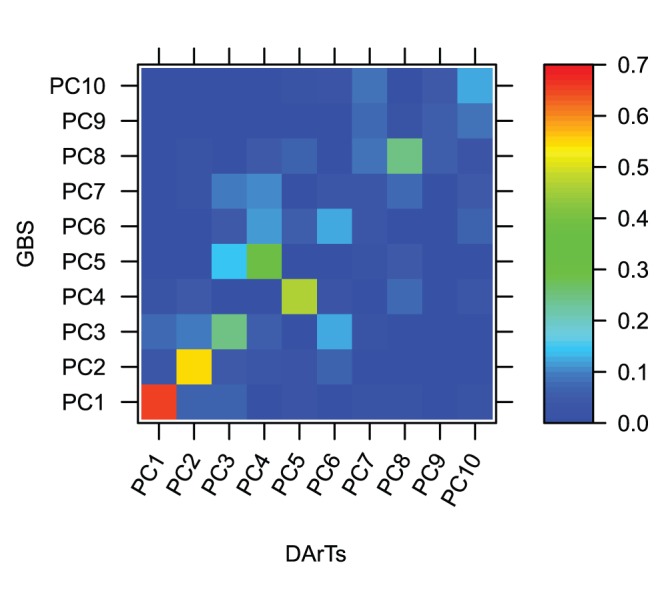
Heatmap of the R^2^ of the eigenvector between the two platform. R^2^ between eigenvectors of the PCA on all DArT markers and eigenvectors of the PCA on all GBS markers after random forest imputation.

A population of 365 soft winter wheat varieties and F_5_–derived advanced breeding lines originating from multiple crosses in the Cornell University Wheat Breeding Program (Ithaca, NY) was analyzed in this study. Lines were genotyped with 5,000 Diversity Array Technology (DArT) markers resulting in 1,544 polymorphic markers. All lines were also genotyped using GBS as described in [18]. The DArT markers had 3.1% missing datapoints and the GBS data had 43.9% missing datapoints for 38,412 markers, where a data point refers to one cell in the marker data matrix. The impact of imputation on the DArT given its low level of missing data was assumed to be marginal [19]. Missing marker data were imputed using random forest [20] as described in [19] separately for the DArT markers and the GBS markers. Using all the markers available for each platform revealed both similarities and differences in the Principal component analysis (PCA) plots (Figure 1). Both PCA plots clearly separated the different large full-sib families present in the data. The first principle component axis explained a similar amount of variation in both analyses, and visually the relationships among lines were similar. In spite of many overall similarities, however, we detected some differences between DArT and GBS PCA. The GBS plot was rotated compared to the DArT markers plot suggesting that the second eigenvector was different between the DArT and GBS markers. There was a scale difference between the DArT markers and the GBS PCAs attributable to the large difference in the number of markers between platforms. Because there are many more markers with GBS, the distances between genotypes appeared larger. The small observed differences in the representation of genetic distances between lines were investigated by analyzing the R^2^ between the eigenvectors of the PCA on all DArT markers with the eigenvectors of the PCA on all GBS markers. This analysis revealed some difference between platforms (Figure 2). If the PCA in both cases were capturing the same patterns and in the same order, the diagonal elements of the heatmap should have had a value of one and all the other cells should have had a value of 0. This was the case for the first few axes because the two first axes between both platforms were correlated (R^2^ of 0.65 and 0.54 respectively). For axes three to five it appeared that both platforms captured a similar pattern but the variance was distributed differently between the axes. For the remaining axes there was very little resemblance between the patterns captured by all GBS and all the DArT markers except for the eighth principal component. This was due to ascertainment bias or to far fewer markers for the DArT markers.

Those differences were further investigated and quantified by using a bootstrap approach to test significance of the differences. 1,544 GBS markers (same number as DArT) were sampled 1000 times and a number of metrics calculated. These tests were used to determine if the difference between DArT and GBS markers was significant, which would indicate that the DArT and GBS markers were drawn from different distributions.

As suggested by Figure 1 results, the big picture of the diversity as measured by the number of identified genotype groups was not significantly different between DArT and GBS (Table 1). The composition of the groups was not compared in the bootstrap approach as there was no simple statistic for comparisons with varying group numbers. Similarly, there were no significant differences in the variances explained by the first eigenvector (P-value 0.176) in both PCA with the bootstrap approach.

**Table 1:** Population genetics parameters computed using the DArT and p-value from the GBS bootstrap.

Parameter	N groups geno	R^2^ 1st PC	R^2^ 2nd PC	*F_st_* (Weir)	Kullback-Leibler divergence	A matrix correlation
**All DArT markers**	8	0.099	0.061	0.24	698.32	0.7
**P-value**	0.205	0.176	0	0	0	0

Number of clusters of lines identified, R^2^ explained by the first two PCA components, *F_st_* corrected for subpopulation size difference. The Kullback-Leibler divergence and the A matrix correlation test the significance of the difference between the A matrix calculated with all the GBS markers and the A matrix based on the DArT markers. Note that the bootstrap P-value does not compare the values obtained with all DArT markers to the value obtained with all GBS.

The *F_st_* (measuring sub-population differentiation) was much higher with the DArT markers than with any bootstrap sample of the GBS markers indicating a stronger apparent population differentiation with the DArT markers. The second eigenvector of the DArT markers PCA captured much less of the total variance than any sample of the GBS markers bootstrap samples. This was an indication of an apparent more complex diversity pattern as captured by the DArT markers.

To measure the information lost when the relationship matrix was calculated using either DArT markers or an equal number of GBS markers, the Kullback-Leibler divergence was used [21] with the same bootstrap approach as previously described. It measured the information lost when the relationship matrix is used to approximate a reference covariance matrix based on all the GBS markers available. The Kullback-Leibler divergence was much higher with the DArT markers than with any bootstrap sample of the GBS markers. Similarly the correlation between the relationship matrix based on DArT markers with the relationship matrix based on all the GBS markers available was much lower than with any bootstrap sample of the GBS markers. This shows that there was a significant difference in the picture of diversity captured by the two marker platforms.

**Figure 3:**
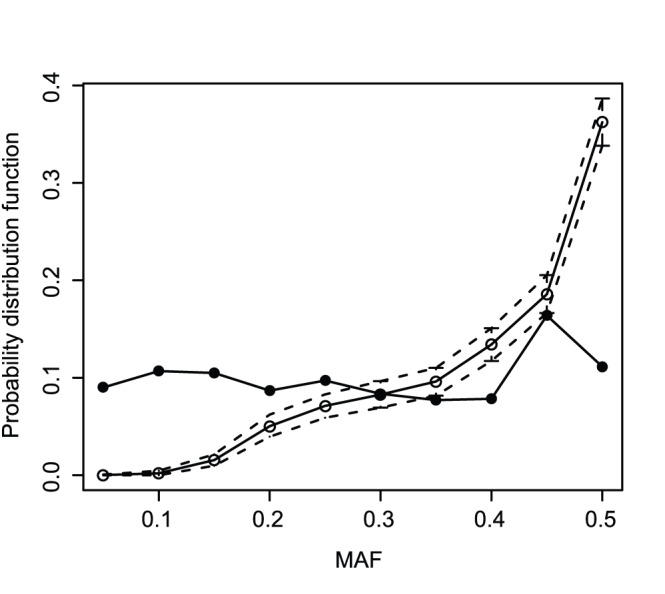
DArT MAF distribution and 95% confidence interval from the GBS bootstrap. The filled circle corresponds to the DArT and the empty circle corresponds to the mean of the 1000 GBS bootstrap samples.

The Minor allele frequency (MAF) distributions of the DArT and of the GBS markers were compared by building a 95% bootstrap confidence interval for quantiles of the GBS bootstrap distribution (Figure 3). If the MAF distribution for the DArT markers were not contained within the 95% confidence interval generated from the GBS bootstrap distribution, it would indicate that the MAF distribution of the DArT is significantly different from the GBS MAF distribution. The graph on Figure 3 shows that DArT markers are outside the GBS confidence interval for a number of MAF bins (Equal intervals of size 0.05). This indicated that the DArT markers MAF distribution significantly differs from the MAF distribution of the GBS markers. The DArT markers show a clear excess of rare variants (MAF below 0.2) compared to the GBS markers and a large deficit of frequent variants (MAF above 0.4). The MAF distribution for all the GBS markers with and without imputation was compared (Figure S1), and the effect of imputation on the MAF distribution was negligible.

**Figure 4:**
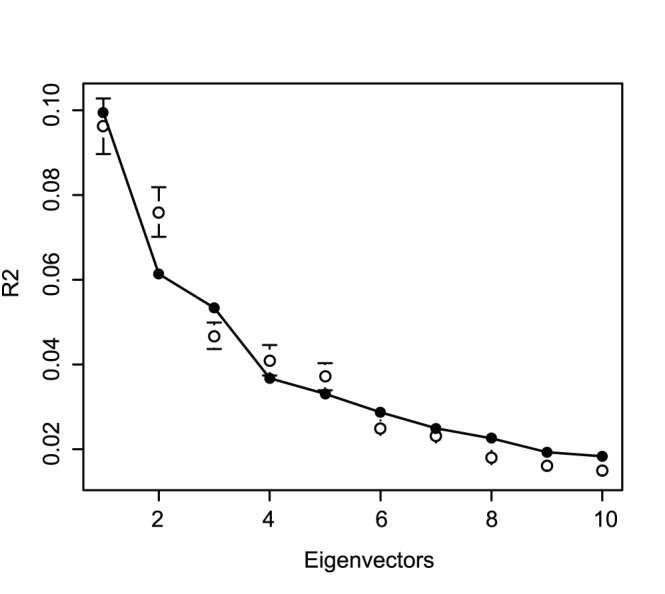
DArT PCA R^2^ and 95% confidence interval from the GBS bootstrap. The filled circle corresponds to the DArT and the empty circle corresponds to the mean of the 1000 GBS bootstrap samples.

A similar confidence interval based on a bootstrap distribution was built for the percent of variance captured by each eigenvector of the PCA and is presented in Figure 4. If the DArT values were outside of the 95% confidence interval it would indicate that the percent of variance captured by each eigenvector of the PCA is significantly different between GBS and DArT. The distribution of variance between eigenvectors for the DArT and the GBS markers was significantly different for every given eigenvector, except the first eigenvector (Figure 4). This also demonstrates that, despite an overall similar main picture (same amount of variance captured by the first component), the diversity picture was significantly different between the GBS and the DArT markers.

### Redundancy Analysis

Bootstrap p-values were calculated to test for a significant difference in marker redundancy between DArT and GBS platforms. A tag SNP selection procedure [22] was used to select subsets of non-redundant markers. In this procedure, pair-wise marker associations were measured using R^2^. The degree of redundancy in the DArT and GBS markers was assessed using R^2^ cutoffs of 0.7, 0.8, and 0.9 for the tag SNP selection procedure (Table 2). All markers were used for this analysis. Bootstrap p-values indicated that there were significantly more redundant DArT than GBS markers, indicating that DArT markers tended to cluster more than the GBS markers. The P-value corresponded to the probability of obtaining the same number or a lower number of non-redundant markers with GBS markers. Similarly, the variance of the Euclidean distance between GBS markers, calculated using their marker scores as predictors, was smaller for all GBS bootstrap samples (mean 27.61) than the DArT markers value (30.86). This indicates that GBS markers were significantly more evenly distributed across the genome than DArT markers. The same analysis was also done with non-imputed markers and gave similar results (Table S1).

**Table 2:** Non-redundant GBS and DArT markers and P-value function of the R^2^ cutoff.

**R^2^ cutoff**	0.9	0.8	0.7
**All GBS**	35,462	31,605	27,197
**All DArT markers**	956	787	699
**P-value**	0	0	0

Note that the bootstrap P-value does not compare the values obtained with all DArT markers to the value obtained with all GBS. Rather the P-value is for the observed DArT markers value on a bootstrap distribution of the GBS markers.

### GS Analysis

Phenotypic data for four traits were analyzed: grain yield, plant height, heading date, and preharvest sprouting (PHS) as described in [15]. Preharvest sprouting is the premature germination of seeds while still attached to the mother plant. As was done previously, a bootstrap approach was used to test for the significance of the difference in cross-validated accuracy between DArT and GBS markers for an equal number of markers. A simple ridge regression Best linear unbiased predictor (BLUP) was used with a 10-fold cross-validation. The cross-validation partition was identical for all analyses. When using the same number of markers, redundant or not, the difference in accuracy was not significant for three out of four traits (based on bootstrap p-values) (Table 3). When using all the GBS markers available, accuracy was higher than with the DArT markers. To demonstrate that inclusion of GBS markers with high levels of missing data was appropriate, subsets of GBS markers were also selected based on a missing data threshold per marker and GS accuracies were computed (Table S2). With variation between traits, accuracies reached a plateau when including markers with a high level of missing data. (Between 15% missing data for heading date and 80% for plant height). This corresponds to a minimum of 4787 GBS markers compared to 1,544 DArT markers available.

**Table 3:** Cross-validated GS accuracy for DArT and GBS and boostrap p-values for the DArT markers.

Trait	All DArTmarkers	non-redundantDArT markers	All GBS	non-redundant GBS	P-value Redundant	P-value non-redundant
YLD	0.36	0.36	0.41	0.39	0.29	0.48
HT	0.48	0.47	0.52	0.53	0.19	0.37
HD	0.30	0.31	0.47	0.43	0.22	0.56
PHS	0.47	0.47	0.57	0.56	0.00	0.06

The cross-validated accuracy is calculated using all DArT markers or all GBS or or with only the non-redundant markers, (YLD: yield, HT: height, HD: heading date, PHS: pre-harvest sprouting). P-values are presented both when all the markers were used for bootstrap and when using only the non-redundant ones for the analysis. To note that the bootstrap P-values do not compare the values obtained with all DArT markers to the value obtained with all GBS.

## Discussion

Our analyses tested for significant differences between DArT and GBS markers when the number of markers was the same. That is, whether the DArT could have been drawn from the same distribution as the GBS markers. Our results indicated that the DArT and GBS marker data yielded significantly different results for several statistics related to diversity. For a number of metrics, it was very clear that the DArT markers were not drawn from the same distribution as the GBS markers. This difference was likely due the ascertainment bias inherent in the DArT markers because DArT markers were discovered and validated on a screening panel independent from the genotyped population while with GBS the marker discovery and genotyping took place at the same time. The analyses showed that the diversity image was distorted using DArT compared to GBS markers for an equal number of markers, even though a largely similar first principle component was captured by both platforms. The difference in eigenvalues R^2^ was significant between platforms indicating an apparently more complex diversity pattern as captured by the DArT markers. This would suggest that DArT markers overestimated the genetic diversity and differentiation in this population compared to the GBS markers. This was a clear indication of ascertainment bias [8]. The significant difference in *F_st_* between platforms was also an indication of ascertainment bias [7].

DArT markers had a significantly different MAF distribution from the GBS markers with an excess of rare variants compared to GBS. The different MAF distribution showed that the DArT polymorphism frequency distribution was quite different from the polymorphism frequency of all the variants in this population. This could be caused by the discovery process, done on an independent screening panel of lines. Only, polymorphisms that were in high frequency in the screening panels are genotyped, while common variants in this breeding population might have been rare or absent in the screening panel, and thus, were not included on the DArT array. We also expect some bias with GBS. If an allele frequency is too low, it will only be read a few times, and likely be discarded by the GBS pipeline as a sequencing error.

Furthermore, we found that greater ascertainment bias in the DArT marker set led to greater redundancy of polymorphisms compared to those of the GBS marker set. This non-random sampling of polymorphisms in the genome (contributing to ascertainment bias) was most likely introduced by the restriction enzymes and screening panels used to develop the DArT array. If the restriction sites are not randomly distributed across the genome, the markers on the DArT array will also be non-randomly distributed, consistent with what we observed. DArT used *TaqI* and *PstI*, while the GBS protocol in this study used *PstI* and *MspI*. The differences between the two protocols go beyond the choice of enzymes as DArT uses arrays of cloned *PstI-PstI* fragments of size 0.4 to 1kb [13] while GBS directly sequences *PstI-MspI* of size between 170 and 350 bp [4]. Because of those differences in protocol it was not possible to test if the observed non-random distribution of the DArT across the genome is due to the choice of restriction enzyme itself or to other constraints of the protocol.

These findings illustrated that the reduced ascertainment bias of GBS compared to DArT markers led to differences in diversity measurements, suggesting that our knowledge of cereals diversity, which is mainly based on DArT markers, should be re-evaluated using GBS or another marker platform with reduced ascertainment bias. As no physically mapped genome sequence that is available is sufficiently anchored for wheat it was not possible to accurately assess the true distribution of polymorphisms across the genome. However, [18] showed that the GBS markers are uniformly spaced across the genome using biparental populations. An unbiased assessment of ascertainment bias would require knowledge of all the polymorphisms in a set of lines for a comparison to those obtained by GBS or any other genotyping method [7]. Some bias might be expected of the GBS platform because the restriction enzymes usually used when creating reduced representation libraries of genomes are methylation sensitive and preferentially target gene rich regions [4]. This is potentially a problem for population genetics studies. However, at this point there is limited ability to correctly sequence and align repetitive regions such that generating markers from repetitive or gene poor regions with GBS is currently a challenge. In addition, as illustrated by Figure 3, identifying rare polymorphisms with GBS is currently a challenge because of confounding with sequencing errors.

Finally, despite differences due to ascertainment bias, GS accuracies between GBS and DArT markers were not significantly different for three traits out of four when the same numbers of markers was used. This difference was still non-significant when using sets of non-redundant markers for the DArT markers and GBS. The difference in accuracy was significant only for PHS suggesting that ascertainment bias had an impact on GS accuracy for that trait only. As DArT are not evenly spaced across the genome, they may under represent areas close to QTLs for the trait leading to a lower accuracy. When using all the GBS markers available, accuracy was higher than with the DArT markers as previously reported in [5]. This can be explained by the much larger number of markers available with GBS compared to the DArT markers. Further analysis revealed that the optimum numbers of markers varied between 4787 and 38120 GBS markers depending on the trait considered (Table S2).

In terms of cost, because both platforms were designed for applications requiring high density genome coverage such as GS and association studies, the cost per genotyped entry is more relevant than cost per marker. Currently, the DArT array used here cost approximately 50 USD per sample while the GBS protocol we used cost less than 20 USD per sample.

This study suggests that the gain in accuracy observed using the GBS compared to the DArT markers was mainly due to a large increase in the number of non-redundant markers available for the analysis. This constitutes further evidence that GBS is the marker platform of choice for further diversity analyses and GS. It also demonstrated that, given a robust imputation strategy, the high amount of missing data in GBS can be handled and imputed even without a reference map or genome sequence for application in GS as pointed out by results in Table 3 and Table S2. As SNP arrays become more widely available in wheat, it would be useful to carry out the same comparison and assess the level of ascertainment bias in SNP arrays compared to GBS. For future studies it is important to understand the quality of a genotyping platform not only based on error rate or polymorphism rate, but also based on the level of ascertainment bias and the number of non-redundant markers.

## Materials and Methods

### Data

A population of 365 soft winter wheat varieties and F_5_–derived advanced breeding lines originating from multiple crosses in the Cornell University Wheat Breeding Program (Ithaca, NY) was analyzed in this study. Lines were genotyped with 5,000 Diversity Array Technology (DArT) markers (Triticarte Pty. Ltd., Yaralumla, ACT, Australia), resulting in 1,544 polymorphic markers. The DArT technology for wheat assayed a reduced representation library of the genome; built on a small subset of genotypes using *PstI* and *TaqI* restriction enzymes. *PstI-PstI* fragments were cloned and the fragments polymorphic between a set of 13 Australian wheat genotypes were printed on an array. Each clone was further validated on a large panel of genotypes for quality and polymorphism [12], [13], [14].

All lines were genotyped using GBS as described in [18]. Briefly, after DNA digestion by two restriction enzymes, *PstI* and *MspI*, barcoded adaptors were ligated and the *PstI-MspI* fragments amplified by PCR (Polymerase chain reaction). Libraries were then pooled to 48-plex and sequenced on Illumina HiSeq2000. The sequencing reads were processed to remove potential sequencing errors and 38,412 SNPs were identified. Detailed protocols can be found in [18] and the latest updates on the GBS approach for wheat can be found on the USDA Wheat Genetics and Germplasm Improvement website (http://www.wheatgenetics.org/research).

Phenotypic data for four traits were analyzed: grain yield, plant height, heading date, and preharvest sprouting (PHS) as described in [15]. Preharvest sprouting is the premature germination of seeds while still attached to the mother plant that decreases grain value and was measured as described by [23], [24]. Phenotypic data were collected from field trials in 2008 and 2009, with three locations per year near Ithaca, NY. Each year, two locations had yield plots (1.26 m by 4 m) and one location had single 1 m rows. All traits were measured in yield trial locations, while PHS, height, and heading date were also measured in single row trials. Each location was arranged in a row-column, augmented design [25] with six check varieties replicated 10 times each.

A two-stage analysis was used to calculate best-linear unbiased estimators (BLUEs) because it was less computationally demanding than a one-stage analysis and has been shown to generate similar results [26]. First, BLUEs were calculated for each trait in each location using a mixed model in ASReml-R [27]. When necessary, the data was corrected for a trend along the rows and the columns of the trial and the covariance of error between neighboring plots modeled [28], [29]. For PHS, an additional random effect of harvest date was included. Second, line BLUEs were calculated across years and locations. The line mean heritability was estimated to be: (yield: 0.29; heading date: 0.73; height 0.77; PHS 0.24). Phenotypic and marker data is available in Data S1.

### Imputation of Genotypic Data

The DArT markers had 3.1% missing datapoints (cells in the marker data matrix) and the GBS data had 43.9% missing datapoints for 38,412 markers. The low level of missing data for the DArT suggested that the impact of imputation would be marginal [19] for this set. Missing marker data were imputed using random forest [20] as described in [19] separately for the DArT markers and the GBS markers. However, to be able to generate certain population genetics statistics a categorical allele call is needed. Thus, instead of random forest regression we used random forest classification to obtain a categorical allele call. Random forest is a machine-learning algorithm that uses an ensemble of decision trees, taking a majority vote of the multiple decision trees to determine a classification or a prediction value for new instances. It is a robust algorithm for classification and regression when there are thousands of input variables. In this study, a majority vote for 100 regression trees was used to impute the missing values for each marker with the RandomForest package [30] in R 2.15.0 [31] using the R package snow for parallelization. For each marker, the training set was the genotypes without missing data for that particular marker. For each classification tree, the algorithm generated a bootstrap sample as the training population. The missing data for that marker were then predicted by each tree and the most frequently called allele was used as the imputed value.

### Diversity Analysis

As a first approach, principal component analysis (PCA) was used to analyze all DArT or GBS data available to look for differences in the representation of the lines. To quantify the differences between the DArT and the GBS platforms, a bootstrap procedure was used. A total of 1,544 imputed GBS markers (same as the number of DArT markers) were sampled and used to compute population genetics statistics. Based on that sample of GBS markers, lines were clustered using the R package *mclust* [32] to identify subpopulations by hierarchical clustering using a parameterized Gaussian mixture model. The Bayesian information criterion (BIC) was used to identify the optimal number of subpopulations as well as the optimal clustering model to use. Based on that subpopulation structure, *F_st_* values were computed to measure the genetic differentiation between subpopulations. *F_st_* measures the fraction of the variance in allele frequencies due to population differentiation. The *F_st_* estimator of [33] which is insensitive to differences in subpopulation sizes was used. The overall gene diversity was computed using the R package hierfstat [34]. To measure the information lost when the relationship matrix was calculated based on sampled GBS markers or DArT markers instead of using all GBS markers, the Kullback-Leibler divergence was used [21]. The Kullback-Leibler divergence is a measure of the difference between two probability distributions. It measured the information lost when the relationship based on sampled GBS markers or DArT markers are used to approximate a reference covariance matrix. The relationship matrix based on all GBS markers was used as a reference. The relationship matrix is equal to *XX^t^*, where *X* is the marker score matrix, of dimensions number of lines by number of markers. Markers ared coded such that {*aa*, *Aa*, *AA*}  =  {−1, 0, 1}. *XX^t^* is also referred to as the realized relationship matrix because it captures relationship between lines, including Mendelian sampling. In the context of the infinitesimal model for quantitative genetics and of genomic selection, the relationship matrix based on markers corresponds to the covariance between genotypes. For multivariate normal distribution and non-singular covariance matrix, the Kullback-Leibler divergence has a simple algebraic formulation. Calculation was carried out using the monomvn R package. The minor allele frequency (MAF) was computed for each marker. Finally, PCA analysis was carried out and the variance captured by each eigenvector calculated for each bootstrap sample. The sampling procedure was repeated 1000 times to generate a bootstrap distribution for the GBS markers.

The same statistics were computed on the entire set of DArT markers. A P-value was computed for the DArT value using the bootstrap GBS distribution to test the null hypothesis that, for an equal number of markers, the diversity picture is the same between GBS and DArT markers. When the P-value was less than 0.05, it showed the presence of significant difference between the two marker platforms for the metric considered and indicated possible ascertainment bias. Finally, PCA analysis was carried out and the variance captured by each eigenvector calculated for each bootstrap sample. The sampling procedure was repeated 1000 times to generate a bootstrap distribution for the GBS markers. The same statistics were computed on the entire set of DArT markers. A P-value was computed for the DArT value using the bootstrap GBS distribution to test the null hypothesis that, for an equal number of markers, the diversity picture is the same between GBS and DArT markers. If the P-value was significant, it showed the presence of significant difference between the two marker platforms and indicated possible ascertainment bias.

### Bootstrap Confidence Interval

To test for significant differences between DArT and GBS platforms in terms of their MAF distributions and the percent of the variance explained by each eigenvector from PCA, a bootstrap confidence interval of the statistics of interest were calculated for the GBS marker set and then compared to that of the DArT set. Specifically, 1000 bootstrap samples of 1,544 GBS markers (same number as DArT) were drawn without replacement, and the statistics of interest were calculated. In the case of the MAF, MAF was computed for each marker and for various MAF bins the proportion of markers belonging to each bin was computed and saved for each bootstrapped sample, generating a distribution of proportions. 95% confidence intervals were then computed using these distributions and the confidence intervals were then compared to the proportion of markers in various MAF bins in the DArT marker set. In the case of percent of variance explained by each eigenvector, for each of the 1000 bootstrapped GBS samples, the percent of the variance explained by each eigenvector was calculated and saved. The distributions of these values were then compared to the percent of the variance explained with each eigenvector using the DArT marker set. Absence of overlap between the DArT values and the 95% confidence intervals of the GBS values indicated significant differences.

### Redundancy Analysis

A similar type of bootstrap analysis was carried out to test for a significant difference in marker redundancy. A tag SNP selection procedure [22] was used to select one SNP for each bin of associated SNPs. Pair-wise SNP associations were measured using R^2^, and SNPs within a bin that had pair-wise R^2^ values greater than or equal to a specified threshold level were considered redundant. The tag SNP within a bin was selected to minimize missing data, and there was no selection for MAF. The R^2^ thresholds of 0.7, 0.8, and 0.9 were compared for the degree of redundancy. The number of tag SNPs resulting from each sample of GBS markers was computed to generate a distribution of the number of non-redundant markers for each R^2^ threshold. The same tag SNP procedure was also applied to the DArT markers to estimate the number that were non-redundant at each threshold level. P-values for each threshold level were computed based on the distributions of the number of non-redundant GBS markers to test the hypothesis that the number of non-redundant markers is significantly different between the DArT and GBS marker sets. As an additional test of difference in marker distribution across the genome, the variance of the Euclidean distance between markers of each GBS sample and for DArT markers was calculated. To calculate this distance between markers, the markers scores of the genotypes were used. A large variance is indicative of an uneven marker distribution across the genome and of marker clustering. A P-value was derived for these statistics using the bootstrap approach.

### GS Analysis

A bootstrap p-value was used to compare the differences in GS accuracy. For each bootstrap, 1,544 imputed GBS markers were sampled and used to compute a realized relationship matrix. A GS model was built using genomic BLUP with the R package rrBLUP [35]. In genomic BLUP the covariance of the lines is constrained by the realized relationship matrix based on markers. A 10-fold cross validation procedure was used keeping the same partition of the folds for every bootstrap. The procedure was repeated for each of the four traits studied. This provided a cross-validated accuracy for each trait and each bootstrap sample. Cross-validated accuracy was also computed using the DArT markers and a P-value derived using 1000 bootstrap samples.

The procedure was repeated after applying the tag SNP selection procedure to the DArT markers using an R^2^ threshold of 0.8. This reduced the number of sampled markers to 787. Tag SNPs for the GBS markers were selected in the same manner, reducing the total number of sampled markers to 31,605. The 10- fold cross-validated accuracies were computed for 1000 samples of 787 GBS markers to obtain a bootstrap distribution of accuracies. The 10-fold cross-validated accuracies were also computed using the 787 non-redundant DArT markers and compared to the GBS accuracy distribution to derive a P-value.

## Supporting Information

Figure S1 **Minor allele frequency histogram for the GBS markers respectively non imputed (A) and imputed (B).**
(EPS)

Table S1 Non-redundant GBS and DArT markers and P-value function of the R^2^ cutoff when the tag SNP procedure was done on the non-imputed data. Note that the bootstrap P-value does not compare the values obtained with all DArT markers to the value obtained with all GBS. Rather the P-value is for the observed DArT markers value on a bootstrap distribution of the GBS markers. For the GBS data, R^2^ was calculated using pairwise complete observations, and if there were fewer than 30 observations overlapping, the R^2^ was considered missing.
(DOC)

Table S2 Cross-validated GS accuracy for subset of GBS markers based on the amount of missing data. Subsets of markers were selected based on a maximum rate of missing data but the imputed data based on the complete marker set was used for the analysis. (YLD: yield, HT: height, HD: heading date, PHS: pre-harvest sprouting). For each missing data threshold, the corresponding number of markers is indicated.
(DOC)

Data S1 Molecular marker and phenotype data.
(ZIP)
