# Supplementary material for: Impact of Marker Ascertainment Bias on Genomic Selection Accuracy and Estimates of Genetic Diversity
Source: PLoS One. 2013 Sep 5;8(9):e74612. doi: 10.1371/journal.pone.0074612 (PMC3764096; doi:10.1371/journal.pone.0074612)
Supplement: Table S1 — Non-redundant GBS and DArT markers and P-value function of the R2 cutoff when the tag SNP procedure was done on the non-imputed data. Note that the bootstrap P-value does not compare the values obtained with all DArT markers to the value obtained with all GBS. Rather the P-value is for the observed DArT markers value on a bootstrap distribution of the GBS markers. For the GBS data, R2 was calculated using pairwise complete observations, and if there were fewer than 30 observations overlapping, the R2 was considered missing. (DOC) [file pone.0074612.s002.doc]

Table S1. Non-redundant GBS and DArT markers and P-value function of the R² cutoff when the tag SNP procedure was done on the non-imputed data. Note that the bootstrap P-value does not compare the values obtained with all DArT markers to the value obtained with all GBS. Rather the P-value is for the observed DArT markers value on a bootstrap distribution of the GBS markers. For the GBS data, R² was calculated using pairwise complete observations, and if there were fewer than 30 observations overlapping, the R² was considered missing.

| **R² cutoff** | 0.9 | 0.8 | 0.7 |
| --- | --- | --- | --- |
| **All GBS** | 18893 | 12019 | 8642 |
| **All DArT markers** | 666 | 722 | 823 |
| **P-value** | 0 | 0 | 0 |
