# Supplementary material for: Impact of Marker Ascertainment Bias on Genomic Selection Accuracy and Estimates of Genetic Diversity
Source: PLoS One. 2013 Sep 5;8(9):e74612. doi: 10.1371/journal.pone.0074612 (PMC3764096; doi:10.1371/journal.pone.0074612)
Supplement: Table S2 — Cross-validated GS accuracy for subset of GBS markers based on the amount of missing data. Subsets of markers were selected based on a maximum rate of missing data but the imputed data based on the complete marker set was used for the analysis. (YLD: yield, HT: height, HD: heading date, PHS: pre-harvest sprouting). For each missing data threshold, the corresponding number of markers is indicated. (DOC) [file pone.0074612.s003.doc]

Table S2. Cross-validated GS accuracy for subset of GBS markers based on the amount of missing data. Subsets of markers were selected based on a maximum rate of missing data but the imputed data based on the complete marker set was used for the analysis. (YLD: yield, HT: height, HD: heading date, PHS: pre-harvest sprouting). For each missing data threshold, the corresponding number of markers is indicated.

| **Maximum % missing** | **0.05** | **0.1** | **0.15** | **0.2** | **0.25** | **0.3** | **0.35** | **0.4** | **0.45** | **0.5** | **0.55** | **0.6** | **0.65** | **0.7** | **0.75** | **0.8** | **0.85** | **0.9** | **All GBS** |
| --- | --- | --- | --- | --- | --- | --- | --- | --- | --- | --- | --- | --- | --- | --- | --- | --- | --- | --- | --- |
| **Number of markers** | **117** | **1965** | **4787** | **7363** | **9904** | **12084** | **14272** | **16423** | **18843** | **21225** | **23854** | **26905** | **30009** | **33432** | **36588** | **38120** | **38368** | **38411** | **38412** |
| YLD | 0.23 | 0.43 | 0.41 | 0.41 | 0.41 | 0.41 | 0.41 | 0.4 | 0.39 | 0.39 | 0.39 | 0.39 | 0.39 | 0.39 | 0.39 | 0.39 | 0.39 | 0.39 | 0.41 |
| HT | 0.36 | 0.51 | 0.52 | 0.51 | 0.52 | 0.52 | 0.51 | 0.51 | 0.52 | 0.52 | 0.52 | 0.52 | 0.52 | 0.52 | 0.52 | 0.53 | 0.53 | 0.53 | 0.52 |
| HD | 0.17 | 0.41 | 0.44 | 0.43 | 0.43 | 0.44 | 0.43 | 0.42 | 0.42 | 0.42 | 0.43 | 0.43 | 0.43 | 0.43 | 0.42 | 0.42 | 0.42 | 0.42 | 0.47 |
| PHS | 0.41 | 0.56 | 0.55 | 0.56 | 0.57 | 0.57 | 0.57 | 0.57 | 0.57 | 0.57 | 0.57 | 0.56 | 0.56 | 0.56 | 0.56 | 0.56 | 0.56 | 0.56 | 0.57 |
